# 

**Published:** 1878-03-20

**Authors:** 


					﻿•
B I
■•H 1
*"7 I
£
'51
> Q I
a ryi
[S
f rH i
•o
O
' H ■
1i-5
t <1
1
J Ph
| £
jO
I
r i—<
| hrt
{
JO
3 cc
i
! HH
■ <!
i h5
O
■t1
: H
S
■ w
! CZ?
M
Ph
Ph
W
cc
■<
• M
t-5
Ph
jVOJL. VIII.
MARCH 20, 1878.
No. 3.
THE SOUTHERN
MEDICAL RECORD.
ft ^.ONTHLY jJoUf^NAL. OF PRACTICAL JMEDICINE.
Terms: $£per annum in advance. Club lyates, 5 copies ;'g. Single copies SOOc.
** quicquiii pieecipies esto ekei is."
a?I
pc I
■o I
H 1
a!
O 1
)— I
t> 1
a i
i
O i
O i
□d
Z !
o
>
Q
’ J
►—<
o
f>
I—(
u—
ft
Sr*
i—I
GO
GO
O
S
Q
ft
ft
ft
o
ESTABLISHED IN 1870.
ATLANTA, GA.:
Sunnt South Steam Publishing House.
Address R. C. WORD, M. D., Managing Editor, Atlanta, Ga.
				

